# Retraction: Introduction of a trinuclear manganese(iii) catalyst on the surface of magnetic cellulose as an eco-benign, efficient and reusable novel heterogeneous catalyst for the multi-component synthesis of new derivatives of xanthene

**DOI:** 10.1039/d3ra90130k

**Published:** 2024-01-05

**Authors:** Pouya Ghamari kargar, Ghodsieh Bagherzade, Hossein Eshghi

**Affiliations:** a Department of Chemistry, Faculty of Sciences, University of Birjand Birjand Iran gbagherzade@gmail.com bagherzade@birjand.ac.ir +98 56 32345192 +98 56 32345192; b Department of Chemistry, Faculty of Science, Ferdowsi University of Mashhad Mashhad Iran

## Abstract

Retraction of ‘Introduction of a trinuclear manganese(iii) catalyst on the surface of magnetic cellulose as an eco-benign, efficient and reusable novel heterogeneous catalyst for the multi-component synthesis of new derivatives of xanthene’ by Pouya Ghamari kargar *et al.*, *RSC Adv.*, 2021, **11**, 4339–4355, https://doi.org/10.1039/D0RA09420J.

The Royal Society of Chemistry, with the agreement of the named author, hereby wholly retracts this *RSC Advances* article due to concerns with the reliability of the data.

The XRD pattern for Fe_3_O_4_ in Fig. 2 contains repeating sections.

The authors provided the raw XRD data for the Fe_3_O_4_ in Fig. 2 of this article and it was found to be identical in a number of different regions to the raw data provided by the authors for CuO in Fig. 4b of ref. 1 and Fig. 3 of ref. 2.

The authors have stated that they outsourced the XRD data collection to an external company.

Given the significance of these concerns, the findings presented in this paper are no longer reliable.

The authors were informed about the retraction of the article. Pouya Ghamari kargar and Ghodsieh Bagherzade have not agreed with the decision.

Signed: Hossein Eshghi

Date: 13^th^ December 2023

Retraction endorsed by Laura Fisher, Executive Editor, *RSC Advances*

## Supplementary Material
